# Is TIMP-1 immunoreactivity alone or in combination with other markers a predictor of benefit from anthracyclines in the BR9601 adjuvant breast cancer chemotherapy trial?

**DOI:** 10.1186/bcr3411

**Published:** 2013-04-09

**Authors:** Alison F Munro, Annette Bartels, Eva Balslev, Christopher J Twelves, David A Cameron, Nils Brünner, John MS Bartlett

**Affiliations:** 1 Transformative Pathology, Ontario Institute for Cancer Research, MaRS Centre, South Tower, 101 College Street, Suite 800, Toronto, ON M5G 0A3, Canada; 2 Section of Pathobiology, Institute of Veterinary Disease Biology, Faculty of Health and Medical Sciences, University of Copenhagen, Denmark; 3 Department of Pathology, Herlev Hospital, Copenhagen, Denmark; 4 University of Leeds and Cancer Research UK Centre, St James' University Hospital, Leeds, LS2 9JT, UK; 5 Edinburgh Cancer Centre, Western General Hospital, Crewe Road South, Edinburgh EH4 2XU, UK; 6 Biomarkers and Companion Diagnostics Group, Edinburgh Cancer Research UK Centre, MRC IGMM, University of Edinburgh, Crewe Road South, Edinburgh, EH4 2XR, UK

## Abstract

**Introduction:**

Predictive cancer biomarkers to guide the right treatment to the right patient at the right time are strongly needed. The purpose of the present study was to validate prior results that tissue inhibitor of metalloproteinase 1 (TIMP-1) alone or in combination with either *HER2 *or *TOP2A *copy number can be used to predict benefit from epirubicin (E) containing chemotherapy compared with cyclophosphamide, methotrexate and fluorouracil (CMF) treatment.

**Methods:**

For the purpose of this study, formalin fixed paraffin embedded tumor tissue from women recruited into the BR9601 clinical trial, which randomized patients to E-CMF versus CMF, were analyzed for TIMP-1 immunoreactivity. Using previously collected data for *HER2 *amplification and *TOP2A *gene aberrations, we defined patients as "anthracycline non-responsive", that is, 2T (TIMP-1 immunoreactive and TOP2A normal) and HT (TIMP-1 immunoreactive and HER2 negative) and anthracycline responsive (all other cases).

**Results:**

In total, 288 tumors were available for TIMP-1 analysis with (183/274) 66.8%, and (181/274) 66.0% being classed as 2T and HT responsive, respectively. TIMP-1 was neither associated with patient prognosis (relapse free survival or overall survival) nor with a differential effect of E-CMF and CMF. Also, TIMP-1 did not add to the predictive value of *HER2, TOP2A *gene aberrations, or to Ki67 immunoreactivity.

**Conclusion:**

This study could not confirm the predictive value of TIMP-1 immunoreactivity in patients randomized to receive E-CMF versus CMF as adjuvant treatment for primary breast cancer.

## Introduction

A number of clinical studies have clearly indicated the superiority of anthracycline-containing chemotherapy over the combination of cyclophosphamide, methotrexate and 5-flourouracil (CMF) in adjuvant treatment of breast cancer [1-3]. However, a significant number of anthracycline treated patients will experience disease recurrence, suggesting that their breast cancers contained tumor cells refractory to adjuvant anthracyclines. Moreover, patients receiving an anthracycline may experience significant toxicity during treatment [4]. With a validated predictive biomarker for anthracycline sensitivity/resistance, it would be possible to direct the toxic adjuvant anthracycline treatment to those patients with the highest likelihood of a treatment benefit while those patients with anthracycline resistant tumors could receive an alternative treatment, for example, a taxane.

A number of studies have suggested that breast cancer patients with HER2 positive tumors, those amplified and/or overexpressing HER2, are those obtaining the greatest benefit from the addition of an anthracycline [5]. Similar data have been presented for the Topoisomerase IIα (TIIα) gene copy number (*TOP2A*) or enzyme, the latter being a target of the anthracyclines [6]. More recently, we have shown that tumor levels of other members of the HER family may be associated with benefit from adjuvant chemotherapy [7]. However, these effects are not substantiated in a recent meta-analysis of multiple trials with data available for HER2 and TOP2A [8]. Emerging data may suggest that novel markers associated with centromeric enumeration probe for chromosome 17 (CEP17) duplication may identify, in part, those patients with anthracycline responsive cancer [9]. However, increasingly, there is recognition of the complex nature of tumor resistance to chemotherapy and the need for multiple markers to stratify patients according to their likelihood of response to chemotherapy.

Tissue inhibitor of metalloproteinase 1 (TIMP-1) protein as determined by immunhistochemistry is another potential molecular marker for anthracycline benefit [10]. Preclinical data linked TIMP-1 to protection against chemotherapy-induced inhibition of apoptosis [11,12], and when applying TIMP-1 immunohistochemistry (IHC) to tissue microarrays (TMAs) from the Danish Breast Cancer Cooperative Group (DBCG) 89D prospective randomized adjuvant trial (cyclophosphamide, epirubicin, 5-fluorouracil (CEF) vs CMF), [10], it was demonstrated that women with breast tumors displaying cancer cell TIMP-1 immunoreactivity had similar benefit from adjuvant chemotherapy regardless of the addition of an anthracycline, while women lacking TIMP-1 immunoreactivity in the cancer cells had a significant improved benefit (increased disease free survival (DFS) and overall survival (OS)) when receiving combination therapy with an anthracycline as compared with women who received treatment with CMF [10]. A subsequent study, including the same DBCG 89D patient cohorts, showed that TIMP-1 immunhistochemistry could be combined with the *HER2 *or *TOP2A *gene copy number forming a biomarker panel which could predict anthracycline benefit in almost double the number of patients as each of these markers could do separately [13]. In a more recent study [14], including patient samples (TMAs) from the Canadian MA5 study in which patients were randomized to receive either CEF or CMF [14], we reported on a substantial reduction in mortality by CEF compared to CMF in patients with a HER2/TIMP-1 or TOP2A/TIMP-1 responsive profile; however, we could not show a similarly significant reduction in recurrence-free survival events, where a benefit of CEF over CMF was found irrespective of TIMP-1 status.

To further test the hypothesis that TIMP-1 in combination with either *HER2 *or *TOP2A *copy number can be used to predict benefit from adjuvant chemotherapy, including an anthracycline, we assessed TIMP-1 immunoreactivity in TMAs obtained from the BR9601 study in which patients were randomized to receive either epirubicin followed by CMF (E-CMF) or CMF alone [2]. On all samples, data on tumor genetic alterations of *HER2 *and *TOP2A *and protein expression of HER2 and Ki67 were available [7].

## Materials and methods

### Patients

The BR9601 study recruited 374 pre- and post-menopausal women with completely excised, histologically confirmed early breast cancer that had a clear indication for adjuvant chemotherapy according to current UK practice, which relies on the Nottingham Prognostic Index for risk stratification. For further details, please see [2]. The patients were randomized between the standard arm of eight cycles of CMF (intravenous (i.v.) cyclophosphamide 750 mg/m^2^, methotrexate 50 mg/m^2 ^and 5-fluorouracil 600 mg/m^2^) every 21 days, and E-CMF (four cycles of 100 mg/m^2 ^of epirubicin every 21 days followed by four cycles of the same CMF regimen). The protocol was approved by central and local ethics committees, and each patient provided written informed consent prior to randomization. The primary outcomes of the BR9601 study were relapse free (RFS) and OS (2). Patients were followed for a mean of 5.30 years (range 2.76 to 8.51 years).

For the current analysis, following approval by the central ethics committee (South East Multi-centre Research Ethics Committee), tissue blocks were retrieved from 321 cases (85.8%) among which 288 cases (77.0%) were applicable for the present study. A total of 30 samples were lost on the TMAs and 3 cases had incomplete follow-up. Triplicate tissue microarrays (TMAs) with 0.6 mm^2 ^cores were constructed using cores taken from the middle of the invasive tumor following review by a pathologist (JST). Duplicate TMAs were used for the purpose of this study.

### Triple color fluorescent *in situ *hybridization (FISH)

FISH was performed using a triple-color probe for *HER2*, Topoisomerase IIα (*TOP2A*) and chromosome 17 (CEP17) (Abbot Molecular, Maidenhead, Berkshire, UK) as previously described [15-17]. Amplifications were defined as gene:chromosome ratios > 1.5 for *TOP2A *and > 2.0 for *HER2*. *TOP2A *deletions were defined as gene:chromosome ratios < 0.8 [17].

### Immunohistochemistry

The TIMP-1 immunohistochemistry procedure used has previously been described in details [18]. In brief, TMAs were deparaffinized in xylene and rehydrated in graded concentrations of ethanol. For antigen retrieval, the sections were microwave treated in citrate buffer pH6 and endogenous peroxidase activity was blocked by hydrogen peroxide. IHC staining for TIMP-1 was performed overnight at 4°C and used the mouse monoclonal antibody, clone VT7 [19] raised against recombinant human TIMP-1 (0.25 μg/ml).

TIMP-1 staining was scored by two experienced observers (AB and EB) blinded to patient identity and outcome. If any TIMP-1 positive cells were evident in the section it was scored as positive, thus following the same procedure as in our previous studies [10,13].

The IHC procedure for Ki67 (clone MIB-1, Dako, Glostrup, Denmark) has been described previously [20]. Staining was scored by a single experienced scorer (AM) blinded to patient identity and outcome and counting the percentage of Ki67 positive cells. For Ki67 13% positivity was used as a cut point for dividing samples into Ki67 low and high, respectively [20], consistent with our previous studies.

### Statistics

The IBM SPSS (v14) statistical software (IBM corporation, Portsmouth, Hampshire, UK) was used for statistical analysis. Kaplan-Meier estimates of survival were used for analysis of RFS and OS. The Cox's proportional hazard model was used to obtain hazard ratios for relapse or death. When comparing outcomes between the treatment arms within the two groups of patients identified by biomarker expression, formal *P*-values were not calculated as in most cases, one group was much smaller than the other. The Cox model was instead used to identify statistically significant interactions between biomarkers (expression or gene alterations) and outcome on the different treatments (treatment by marker effect), in models that also included biomarker status (marker effect) and treatment, as covariates.

RFS was calculated from the date of randomization to the date of relapse or the date last seen. OS was calculated from the date of randomization to the date of breast cancer specific death or the date last seen.

## Results

There were no significant differences in patient baseline characteristics between the overall BR9601 trial population (*n *= 374) and the population (*n *= 288) included in this biomarker study (Table 1). Of 288 patients included in the current study, 96 (33.3%) relapsed and 78 (27.1%) died during the follow-up period. A survival analysis of these 288 patients confirmed the advantage of E-CMF over CMF observed in the main trial [2] with HR: 0.57; 95% CI 0.37 to 0.86; *P *= 0.006 and HR: 0.64; 95% CI 0.41 to 1.01; *P *= 0.05 for RFS and OS, respectively. All subsequent analyses are restricted to these 288 cases, or sub-sets thereof.

**Table 1:** Patient/tumor characteristics from the BR9601 trial and samples retrieved for the current study

	*BR9601*	*TIMP-1 Analysis*
Number	374	288
Age	50.9 (44.7 to 56.6)	50.5 (45.0 to 57.0)
E-CMF (1)	183 (48.9%)	140 (48.6%)
CMF (2)	191 (51.1%)	148 (51.4%)
Tumor size (median)	23 (17 to 30)	25 (17 to 30)
Positive nodes	2 (1 to 4)	3 (1 to 4)
Grade	3 (2 to 3)	3 (2 to 3)
NPI	5.30 (4.50 to 5.61)	5.21 (4.50 to 5.60)
ER +ve	202 (54.0%)	158 (54.9%)
ER -ve	119 (31.8%)	96 (33.3%)
ER Unk	53 (14.2%)	34 (11.8%)

### TIMP1 immunoreactivity

**Figure 1:**
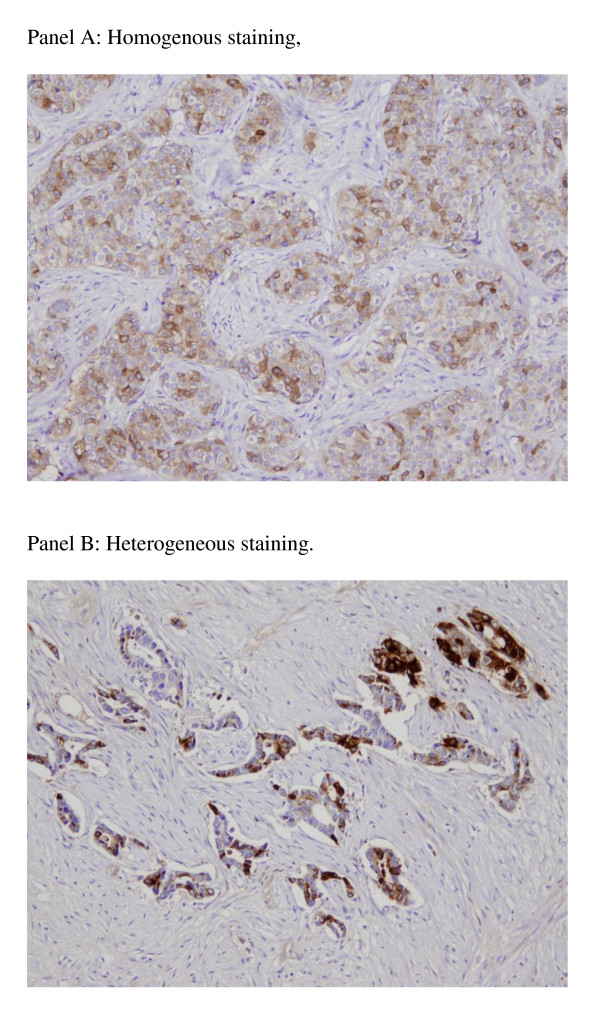
**Illustrative figures of TIMP1 staining**. Panel **A**: Homogenous staining. Panel **B**: Heterogeneous staining.

Successful TIMP-1 staining was achieved for 291/321 cases. Three cases were lost to follow-up and excluded from subsequent analysis. Some TIMP-1 positive tumors displayed a homogenous staining, while others presented with a heterogenous staining pattern (Figure 1). Of the 288 cases available for analysis: 42% of the tumors had one or two TIMP-1 positive cores while 58% presented with two TIMP-1 negative cores.

### *HER2, TOP2A *and Ki67

Results for *HER2 *and *TOP2A *gene copy analyses and Ki67 protein analysis have been previously presented and are summarized in Table 2.

**Table 2:** *HER2 *and *TOP2A *gene status and Ki67 protein expression in the BR9601 cohort used for this study

		*Amplified*	*Deleted*	*Normal*
***HER2 *FISH**	274/288 (95.1%)	62 (22.6%)		212 (77.4%)
***TOP2A *FISH**	274/288 (95.1%)	25 (9.1%)	48 (17.5%)	201 (73.4%)
		***High***	***Low***	
**Ki67**	284/288 (98.6%)	120 (42.3%)	164 (57.7%)	

### Associations between TIMP-1 and Ki67 immunoreactivity and *HER2 *and *TOP2A *gene copy numbers and RFS and OS

The prognostic significance of each of the biological markers included in this study was first tested on the entire included patient cohort (*n *= 288), irrespective of their allocated adjuvant chemotherapy. Exploratory analysis of these markers (minus TIMP-1) showed the results to be similar in this cohort and the 321 patients included in the original biomarker study [7]. TIMP-1 immunoreactivity was not significantly associated with RFS (HR = 0.83; 95% CI: 0.55 to 1.24; *P *= 0.36) or OS (HR = 0.69; 95% CI: 0.43 to 1.09; *P *= 0.11). However, TIMP-1 positivity was significantly associated with an increased number of positive lymph nodes (*P *= 0.01), ERα positive disease (*P *= 0.004), and decreased proliferation (measured by Ki67; *P *= 0.004; Table 3).

**Table 3:** Associations between TIMP-1, Ki-67, *HER2 *and *TOP2A*

		*TIMP-1 Negative*	*TIMP-1 Positive*	*P-value*
**Ki67**	Low	83 (50.6%)	81 (67.5%)	0.004
	High	81 (49.4%)	39 (32.5%)	
***HER2***	Normal*	119 (75.8%)	93 (79.5%)	0.470
	Amplified	38 (24.2%)	24 (20.5%)	
***TOP2A***	Deleted	32 (20.4%)	16 (13.7%)	0.310
	Normal	110 (70.1%)	91 (77.8%)	
	Amplified	15 (9.6%)	10 (8.5%)	

Normal includes samples with HER2 deletions and HER2 normal samples.

Tumors were classified as 2T responsive (*TOP2A *abnormal and/or TIMP-1 negative) and 2T non-responsive (*TOP2A *normal and TIMP-1 immunoreactive) [13]. Using this definition, 183/274 (66.8%) of tumors were classed as 2T responsive and 91/274 (33.2%) as 2T non-responsive. There was no significant difference in RFS (HR = 0.91; 95% CI: 0.585 to 1.417; *P *= 0.68) and OS (HR = 0.773; 95% CI: 0.470 to 1.271; *P *= 0.30) between patients with the 2T responsive profile when compared to those with the non-responsive profile. A 2T responsive profile was associated with ERα negative disease (*P *= 0.0001), increased pathological grade (*P *= 0.005), and increased proliferation (*P *= 0.001).

On the basis of TIMP-1 immunoreactivity and *HER2 *gene copy numbers, 181 (66%) of tumors were classified as HT responsive (*HER2 *amplified and/or TIMP-1 negative) and 93 (34%) as HT non-responsive (*HER2 *negative and TIMP-1 immunoreactive). Patients who had an HT responsive profile had a significantly decreased RFS (HR = 1.64; 95% CI: 1.035 to 2.6; *P *= 0.037) and OS (HR: 2.08; 95% CI: 1.21 to 3.56; *P *= 0.007) when compared to those with a non-responsive profile. A HT responsive profile was associated with ERα negative disease (*P *= 0.0002), increased pathological grade (*P *= 0.003), and increased proliferation (*P *= 0.0002).

### Treatment by marker analysis of the influence of TIMP-1, *TOP2A *and *HER2 *on RFS and OS benefits of E-CMF over CMF

Subsequent analyses focused on possible differential effects of the expression of these markers on RFS and OS between patients receiving E-CMF and those treated with CMF alone. The results for *HER2 *and *TOP2A *gene copy number counts have been published previously [7].

**Figure 2:**
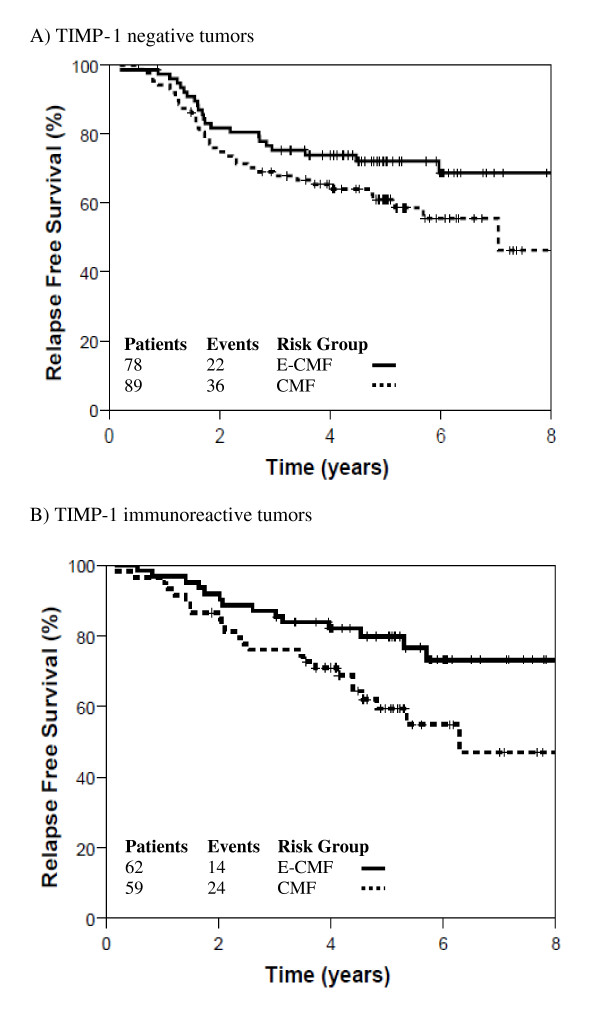
**Relapse free survival for E-CMF (solid line) versus CMF (dashed line)**. Panel **A**: TIMP-1 negative tumors. Panel **B**: TIMP-1 immunoreactive tumors.

Hazard ratios for TIMP-1 immunoreactivity and associated profiles between patients receiving E-CMF and CMF alone are summarized in Table 4. Treatment by marker (TxM) HRs demonstrates that there is no evidence that lack of TIMP-1 immunoreactivity alone or in combination with *TOP2A *or *HER2 *gene aberrations is predictive of anthracycline benefit. In patients with TIMP-1 immunoreactivity there is a trend towards an increase in RFS (HR = 0.48; 95% CI: 0.24 to 0.93; Figure 2A) and OS (HR = 0.46; 95% CI: 0.21 to 1.00) in patients treated with E-CMF compared to CMF alone. However, a similar trend for RFS (HR = 0.64; 95% CI: 0.38 to 1.10; Figure 2B) and OS (HR = 0.78; 95% CI: 0.45 to 1.38) was apparent in patients whose tumors were negative for TIMP-1 suggesting the trend is associated with benefit from treatment irrespective of the immunoreactivity of TIMP-1.

**Table 4:** Unadjusted HR estimates of the treatment by marker effects

		*RFS*			*OS*		
		**HR**	**95% CI**	**TxM (HR)**	**HR**	**95% CI**	**TxM (HR)**
**TIMP-1**	Negative (*n *= 167; 58%)	0.642	0.377 to 1.091	1.311	0.784	0.447 to 1.376	1.667
	Positive (*n *= 121; 42%)	0.478	0.247 to 0.927	(0.562 to 3.056)	0.461	0.213 to 1.002	(0.641 to 4.339)
**2T Profile**	Responsive (*n *= 183; 66.8%)	0.613	0.365 to 1.029	1.022	0.450	0.188 to 1.080	1.579
	Non-responsive (*n *= 91; 33.2%)	0.582	0.277 to 1.221	(0.415 to 2.520)	0.739	0.428 to 1.277	(0.565 to 4.411)
**HT Profile**	Responsive (*n *= 181; 66.1%)	0.658	0.400 to 1.082	0.902	0.783	0.464 to 1.320	0.577
	Non-responsive (*n *= 93; 33.9%)	0.531	0.235 to 1.200	(0.347 to 2.346)	0.384	0.141 to 1.046	(0.186 to 1.788)

## Discussion

This study, which included 288/374 of the patients enrolled in the original BR9601 adjuvant study [2], failed to confirm the predictive value of TIMP-1 protein measurements for anthracycline benefit [10,13] either as a stand-alone biomarker or in combination with *HER2 *or *TOP2A *gene aberrations. It should be mentioned, however, that in the original biomarker study of the BR9601, neither *HER2 *nor *TOP2A *gene aberrations were associated with benefit from anthracyclines [7].

Although only 288 of the original 374 patient samples were available for this study, survival analyses confirmed the benefit from the addition of an anthracycline to CMF versus CMF alone in this subset of patients. Moreover, when analyzing the associations between patient outcome and *HER2 *and *TOP2A *gene copy number and Ki-67 protein in the 288 patients, similar results were obtained as reported previously [7]. It thus appears that these 288 patients are representative for the initial study population.

The TIMP-1 immunostaining was performed using a validated anti-TIMP-1 monoclonal antibody and a strict protocol [18] and the scoring of TIMP-1 positivity/negativity was performed as previously described [10]. However, among the 288 patients 42% were found positive for TIMP-1, which is much less than what has previously been reported: in the DBCG 89D patient cohort 75% of the cases were TIMP-1 positive [10] and a similar distribution between TIMP-1 positivity/negativity was seen in the MA5 study [14]. While TIMP-1 immunoreactivity is often heterogeneous [10] and smaller TMA might impact on results, the cores used here were similar to those used in the MA5 study. Also while in the DBCG 89D study [10], the cores were taken from the invasive front of the tumors while in the BR9601 and MA5 study sampling was focused on tumor rich areas. TIMP-1 immunoreactivity was significantly associated with lower Ki67 immunoreactivity, suggesting that TIMP-1 positive tumors have a lower rate of proliferation, which in turn might result in reduced sensitivity to chemotherapy. Conversely, in the present study in which TIMP-1 positive tumors had a non-significant increased benefit from the E-CMF combination as compared to CMF alone is not explained by increased proliferation of these tumors as evaluated by the Ki67 staining.

An alternative explanation for the discordant results between the present study and our previous studies [10,13,14] is that the interaction depends on the regimens and/or the patient populations studied. The anthracycline regimens in the DBCG and the BR9601 studies are not identical, with a greater total dose and duration of epirubicin treatment in the DBCG study [3] as compared to BR9601 [2]. Furthermore, the DBCG trial recruited only pre-menopausal node-positive women, and patients did not receive adjuvant endocrine therapy. Conversely, in BR9601, both pre- and post-menopausal women were recruited, including 15% node negative tumors, and all ER positive cases were to receive five years of tamoxifen. Another noteworthy disparity between the trials was the higher percentage of ER positive patients recruited into BR9601 (54.9%) compared to the DBCG trial (33.7%). While these differences might explain the discordant results, there is no obvious *a priori *explanation as to why they should.

## Conclusions

In conclusion, this validation study of TIMP-1 breast cancer cell immunoreactivity as a predictive biomarker for adjuvant anthracycline benefit did not support the use of this marker to select patients for anthracycline treatment. Moreover, this study could not confirm any additive predictive value by combining TIMP-1 immunoreactivity with results on *HER2 *and *TOP2A *FISH analyses.

## Abbreviations

CEF: Cyclophosphamide, epirubicin, 5-fluorouracil; CEP17: Centromeric enumeration probe for chromosome 17; CMF: Cyclophosphamide, methotrexate, 5-fluorouracil; DFS: Disease free survival; E-CMF: Epirubicin-CMF; ER: Estrogen receptor; FISH: Fluorescent *in situ *hybridization; HR: Hazard ratio; IHC: Immunohistochemistry; i.v.: Intravenous; OS: Overall survival; RFS: Relapse free survival; TIIα: Topoisomerase IIα; TIMP-1: Tissue inhibitor of metalloproteinase 1; TMA: Tissue microarray.

## Competing interests

The authors declare that they have no competing interests.

## Authors' contributions

JB, AM, DC, CT and NB designed the study. JB, DC, CT and AM collected the tissue samples, established the database and performed the statistical analysis. EB and AB performed and scored the TIMP-1 immunhistochemistry. AM, JB and NB interpreted the data and drafted the manuscript. All authors have read and approved the manuscript for publication.
